# Characteristics of HIV-1 Serodiscordant Couples Enrolled in a Clinical Trial of Antiretroviral Pre-Exposure Prophylaxis for HIV-1 Prevention

**DOI:** 10.1371/journal.pone.0025828

**Published:** 2011-10-05

**Authors:** Andrew Mujugira, Jared M. Baeten, Deborah Donnell, Patrick Ndase, Nelly R. Mugo, Linda Barnes, James D. Campbell, Jonathan Wangisi, Jordan W. Tappero, Elizabeth Bukusi, Craig R. Cohen, Elly Katabira, Allan Ronald, Elioda Tumwesigye, Edwin Were, Kenneth H. Fife, James Kiarie, Carey Farquhar, Grace John-Stewart, Lara Kidoguchi, Dana Panteleeff, Meighan Krows, Heena Shah, Jennifer Revall, Susan Morrison, Lisa Ondrejcek, Charlotte Ingram, Robert W. Coombs, Jairam R. Lingappa, Connie Celum, for the Partners PrEP Study Team

**Affiliations:** 1 Department of Global Health, University of Washington, Seattle, Washington, United States of America; 2 Department of Medicine, University of Washington, Seattle, Washington, United States of America; 3 Department of Epidemiology, University of Washington, Seattle, Washington, United States of America; 4 Department of Laboratory Medicine, University of Washington, Seattle, Washington, United States of America; 5 Department of Pediatrics, University of Washington, Seattle, Washington, United States of America; 6 Statistical Center for HIV/AIDS Research and Prevention, Fred Hutchinson Cancer Research Center, Seattle, Washington, United States of America; 7 Department of Obstetrics & Gynecology, University of Nairobi & Kenyatta National Hospital, Nairobi, Kenya; 8 Centers for Disease Control and Prevention, Entebbe, Uganda; 9 The AIDS Support Organization, Kampala, Uganda; 10 Centers for Disease Control and Prevention, Atlanta, Georgia, United States of America; 11 Center for Microbiology Research, Kenya Medical Research Institute, Nairobi, Kenya; 12 Department of Obstetrics, Gynecology and Reproductive Sciences, University of California San Francisco, San Francisco, California, United States of America; 13 Infectious Disease Institute, Makerere University, Kampala, Uganda; 14 Department of Medicine, University of Manitoba, Winnipeg, Canada; 15 Kabwohe Clinical Research Center, Kabwohe, Uganda; 16 Department of Reproductive Health, Moi University, Eldoret, Kenya; 17 Department of Medicine, Indiana University, Indianapolis, Indiana, United States of America; 18 DF/Net Research, Inc., Seattle, Washington, United States of America; 19 Department of Molecular Medicine and Hematology, University of the Witwatersrand, and Contract Laboratory Services (CLS), Wits Health Consortium, Johannesburg, South Africa; UCL Institute of Child Health, University College London, United Kingdom

## Abstract

**Introduction:**

Stable heterosexual HIV-1 serodiscordant couples in Africa have high HIV-1 transmission rates and are a critical population for evaluation of new HIV-1 prevention strategies. The Partners PrEP Study is a randomized, double-blind, placebo-controlled trial of tenofovir and emtricitabine-tenofovir pre-exposure prophylaxis to decrease HIV-1 acquisition within heterosexual HIV-1 serodiscordant couples. We describe the trial design and characteristics of the study cohort.

**Methods:**

HIV-1 serodiscordant couples, in which the HIV-1 infected partner did not meet national guidelines for initiation of antiretroviral therapy, were enrolled at 9 research sites in Kenya and Uganda. The HIV-1 susceptible partner was randomized to daily oral tenofovir, emtricitabine-tenofovir, or matching placebo with monthly follow-up for 24–36 months.

**Results:**

From July 2008 to November 2010, 7920 HIV-1 serodiscordant couples were screened and 4758 enrolled. For 62% (2966/4758) of enrolled couples, the HIV-1 susceptible partner was male. Median age was 33 years for HIV-1 susceptible and HIV-1 infected partners [IQR (28–40) and (26–39) respectively]. Most couples (98%) were married, with a median duration of partnership of 7.0 years (IQR 3.0–14.0) and recent knowledge of their serodiscordant status [median 0.4 years (IQR 0.1–2.0)]. During the month prior to enrollment, couples reported a median of 4 sex acts (IQR 2–8); 27% reported unprotected sex and 14% of male and 1% of female HIV-1 susceptible partners reported sex with outside partners. Among HIV-1 infected partners, the median plasma HIV-1 level was 3.94 log_10_ copies/mL (IQR 3.31–4.53) and median CD4 count was 496 cells/µL (IQR 375–662); the majority (64%) had WHO stage 1 HIV-1 disease.

**Conclusions:**

Couples at high risk of HIV-1 transmission were rapidly recruited into the Partners PrEP Study, the largest efficacy trial of oral PrEP. (ClinicalTrials.gov NCT00557245)

## Introduction

Pre-exposure prophylaxis (PrEP), in which an HIV-1 susceptible individual uses oral or topical antiretroviral medications for prevention of HIV-1 acquisition, is a promising biomedical HIV-1 prevention strategy under investigation among diverse at-risk populations worldwide [1]. Current efficacy studies have chosen PrEP agents based on the antiretroviral medication tenofovir, as a vaginal gel or as oral tenofovir disoproxil fumarate (TDF) and co-formulated emtricitabine/tenofovir disoproxil fumarate (FTC/TDF). Non-human primate studies have found that oral and topical tenofovir-based PrEP can provide high levels of protection (70–100%) when given prior to systemic or mucosal simian human immunodeficiency virus (SHIV) challenge [2], [3], [4], [5], [6]. Recent clinical trials have demonstrated promising efficacy of PrEP in decreasing HIV-1 acquisition risk in populations receiving standard prevention services. Coitally-associated use of a 1% tenofovir vaginal gel decreased the risk of HIV-1 acquisition among heterosexual women by 39% [7]. Among men who have sex with men (MSM), oral FTC/TDF resulted in a 44% reduction in HIV-1 incidence [8]. Both of these studies also showed increased levels of protection with better adherence to PrEP. In contrast, a trial of daily oral FTC/TDF among high-risk African women was terminated early for lack of efficacy, with equal number of infections in the active and placebo arms [9].

HIV-1 susceptible individuals within HIV-1 serodiscordant partnerships (in which one of the partners is infected with HIV-1 and the other is HIV-1 susceptible) are at high risk of HIV-1 acquisition [10]. A substantial proportion of new HIV-1 infections in sub-Saharan Africa occur within stable heterosexual HIV-1 serodiscordant couples [11], [12]. Recent data from HPTN 052, a randomized, placebo-controlled, clinical trial conducted among 1,763 HIV-1 serodiscordant couples to assess HIV-1 prevention and clinical benefits of immediate (CD4 350–550 cells/µL) versus delayed (CD4<250 cells/µL) antiretroviral therapy found that immediate initiation reduced HIV-1 transmission within the partnership by 96% [13]. However, early initiation of therapy would not address HIV-1 infections acquired from outside partners, which have been seen in up to 30% of new infections occurring within serodiscordant couples in prior prospective studies [14]. Therefore, complementary HIV-1 prevention interventions are needed, such as PrEP, which may reduce risk of HIV-1 acquisition in HIV-1 uninfected persons who have a known HIV-1 infected partner who is not on antiretroviral therapy or who have risk from outside partnerships of unknown HIV-1 serostatus.

We are conducting the Partners PrEP Study, a randomized clinical trial of daily oral TDF and FTC/TDF PrEP to decrease HIV-1 acquisition within HIV-1 serodiscordant heterosexual couples, which is the only efficacy trial of PrEP in this high-risk population. The potential for differential safety, cost and efficacy for TDF and FTC/TDF as oral PrEP argues for evaluating both as potential PrEP agents, ideally in parallel in a single clinical trial. Unique to the Partners PrEP study is the inclusion of heterosexual men, the potential to evaluate the efficacy of PrEP by level of HIV-1 exposure (i.e., since HIV-1 plasma RNA concentrations are measured at enrollment in the HIV-1 infected partner), and the ability to assess whether drug resistance in breakthrough infections is acquired or transmitted. Here, we describe the design of the trial and baseline characteristics of the Partners PrEP Study cohort.

## Methods

### Ethics statement

The University of Washington Human Subjects Review Committee and ethics review committees at collaborating institutions at each of the study sites (Indiana University, Kenya Medical Research Institute, Kenyatta National Hospital, Moi University Teaching and Referral Hospital, Uganda National Council of Science and Technology, Uganda Virus Research Institute, United States Centers for Disease Control and Prevention, University of California San Francisco) approved the study protocol. All participants provided written informed consent.

### Study design

**Figure 1:**
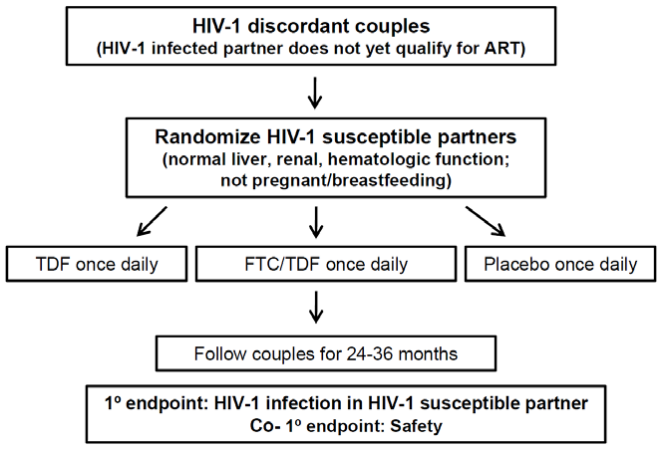
Study Schema.

The Partners PrEP Study is a phase III, multi-site, randomized, double-blind, parallel-arm, placebo-controlled trial of daily oral TDF or FTC/TDF PrEP for the prevention of HIV-1 acquisition by HIV-1 susceptible members of HIV-1 serodiscordant couples (ClinicalTrials.gov number NCT00557245). HIV-1 susceptible partners are assigned in a 1∶1∶1 ratio to one of three study arms: TDF, FTC/TDF, or placebo (Figure 1). The primary study objectives are to: 1) assess the efficacy of TDF and FTC/TDF PrEP for preventing HIV acquisition in the susceptible partner and 2) evaluate safety, each compared against the common placebo arm. The study is end-point driven – i.e., 191 total HIV-1 seroconversion endpoint events (147 per each comparison of TDF or FTC/TDF versus placebo) were determined prior to the trial to be necessary to achieve 80% power with a one-sided alpha of 0.025. The trial's primary objective is to evaluate effectiveness of TDF and/or FTC/TDF PrEP for HIV-1 prevention, under the assumptions that PrEP will decrease HIV-1 risk by 60% (the alternative hypothesis, consistent with other trials of oral and topical tenofovir-based PrEP, which were designed to detect reductions in HIV-1 risk of 50–70% [1]), the lower bound of the 95% confidence interval for efficacy will exclude 30% reduced HIV-1 risk (the null hypothesis for sample size calculations), and that 10% of study follow-up time in HIV-1 susceptible women assigned to the active study arms will be unexposed to PrEP due to protocol-mandated study drug hold during pregnancy. A sample size of approximately 4700 HIV-1 serodiscordant couples (1566 in each treatment arm) was determined to be sufficient to achieve the target number of study endpoints, with 24–36 months of follow-up per participant, assuming up to 5% loss to follow-up per year and an anticipated HIV-1 incidence of 2.75 per 100 person-years in the placebo arm, as was observed in a recent cohort of African HIV-1 serodiscordant heterosexual couples [14]. Gilead Sciences (Foster City, CA) donated TDF, FTC/TDF, and placebo tablets for the study but did not have any role in the development of the study protocol.

### Recruitment and study eligibility criteria

The study is being conducted at nine research sites in Kenya (Eldoret, Kisumu, Nairobi, and Thika) and Uganda (Jinja, Kabwohe, Kampala, Mbale, and Tororo). HIV-1 serodiscordant couples were recruited from a variety of sources, including HIV-1 voluntary counseling and testing (VCT) centers, antenatal clinics and programs for prevention of mother-to-child HIV-1 transmission, referral from HIV-1 care providers including those performing testing of partners of known HIV-1 infected individuals engaged in HIV-1 care programs who were not eligible for ART, and community promotion activities for couples' VCT. Study recruitment occurred between July 2008 and November 2010. The inclusion and exclusion criteria are detailed in Table 1.

**Table 1:** Study Inclusion and Exclusion Criteria.

	HIV-1 susceptible partners		HIV-1 infected partners	
**Inclusion Criteria**	HIV-1 susceptible based on parallel negative HIV-1 rapid tests, at study screening and enrollment visits		HIV-1 infected based on positive EIA	
	Age ≥18 and ≤65 years		Age ≥18 years	
	Sexually active	defined as six or more episodes of vaginal intercourse with the HIV-1 infected study partner in the 3 previous months	Sexually active	defined as six or more episodes of vaginal intercourse with the HIV-1 infected study partner in the 3 previous months
		plan to remain in the relationship for the study period		plan to remain in the relationship for the study period
	Adequate renal function	creatinine clearance ≥60 ml/min, and	CD4 cell count ≥250 cells/µL	
		serum creatinine ≤1.3 mg/dL (men) or serum creatinine ≤1.1 mg/dL (women)		
	Adequate hepatic function	total bilirubin ≤1.5× upper limit of normal, and	No history of any clinical AIDS-defining diagnoses and not otherwise meeting national guidelines for initiation of antiretroviral therapy. In July 2010, Kenya guidelines increased CD4 eligibility for initiation of antiretroviral therapy from <200 to <350 cells/µL and study eligibility was determined based on those updated guidelines	
		hepatic transaminases (ALT and AST) <2× upper limit of normal		
	Adequate hematologic function	absolute neutrophil count >1,300/mm^3^	Able and willing to provide adequate locator information for study retention purposes	
		platelets>125,000/mm^3^		
		hemoglobin>11 g/dL		
	No evidence of chronic active hepatitis B infection	negative hepatitis B surface antigen test	Able and willing to provide written informed consent	
	Able and willing to provide adequate locator information for study retention purposes			
	Able and willing to provide written informed consent			
**Exclusion Criteria**	Current pregnancy or planning to become pregnant during the study period		Enrolled in an HIV-1 treatment trial	
	Current breastfeeding		Current use of antiretroviral therapy	
	Repeated positive (≥1+) urine dipstick tests for glycosuria or proteinuria			
	Ongoing therapy with: antiretroviral therapy; metformin; aminoglycoside antibiotics; amphotericin B; cidofovir; systemic chemotherapeutic agents; other agents with significant nephrotoxic potential			
	History of pathological bone fractures not related to trauma			
	Enrolled in another HIV-1 vaccine or prevention trial			
	Known plans to re-locate or travel away from the study site for more than two consecutive months during study period			

### Study procedures

HIV-1 susceptible participants are followed monthly, for HIV-1 serologic testing, pregnancy testing (for women), provision of study medication, adherence counseling, and behavioral risk assessment; study product is discontinued in those who acquire HIV-1 or during pregnancy. HIV-1 infected partners are followed quarterly, with quarterly monitoring of HIV-1 clinical status and 6-monthly CD4 counts. HIV-1 infected partners who become eligible for initiation of antiretroviral therapy according to the national guidelines of Kenya and Uganda during follow-up are actively counseled to initiate treatment, referred, and linked into care at local HIV-1 clinics. Pregnant HIV-1 infected women are actively referred for antenatal care, including services for prevention of mother-to-child HIV-1 transmission. All participants receive regular individual and couples HIV-1 counseling, condoms, contraception counseling and provision, risk reduction counseling, and syndromic treatment for sexually transmitted infections according to WHO guidelines. Uncircumcised HIV-1 susceptible men are counseled about and referred for circumcision.

### Laboratory procedures

Protocol-specified laboratory assays performed at study sites are detailed in Table 2. For HIV-1 infected partners, plasma HIV-1 RNA concentrations from samples collected at enrollment were quantified in batch testing at the University of Washington using the Abbott Real-Time HIV-1 RNA assay (Abbott); the limit of quantification was 80 copies/mL. Sites were enrolled in External Quality Assurance (EQA) programs for protocol-specified laboratory tests.

**Table 2:** Study Site Laboratory Assays.

	Kenya				Uganda				
	Eldoret	Kisumu	Nairobi	Thika	Jinja	Kampala	Kabwohe	Mbale	Tororo
**HIV-1 rapids**	Determine HIV 1/2 (Abbott/Inverness Medical)				Determine HIV 1/2 (Abbott/Inverness Medical)				
	Unigold (Trinity Biotech)		Bioline (Standard Diagnostics)		Unigold (Trinity Biotech)			HIV 1/2 STAT-PAK (Chembio Diagnostic Systems)	
**HIV-1 enzyme immunoassays** *	Vironostika HIV Ag/Ab 4^th^ gen (bioMérieux)				Vironostika HIV Ag/Ab 4^th^ gen (bioMérieux)		Vironostika HIV Uni-Form II plus O – 3^rd^ gen (bioMérieux)		
	Murex HIV Ag/AB Combo 4^th^ gen (Abbott Murex)				BioRad HIV 1/2 (Bio-Rad Laboratories)			Murex HIV 1.2.0 AB 3^rd^ gen (Abbott Murex)	
**Urine dipstick (protein, glucose)**	Roche Combur 3 (Roche Diagnostics)	Combina 10 M (Human Diagnostics)	Roche Combur 3 (Roche Diagnostics)		Roche Combur 3 (Roche Diagnostics)			Urine-2 (Cypress Diagnostics)	Combina 11 S (Human Diagnostics)
**Urine pregnancy**	QuickVue (Quidel Corporation)				QuickVue (Quidel Corporation)		Hexagon (Human Diagnostics)		Acon (ACON Laboratories)
**CD4**	BD FACSCalibur (BD Biosciences)	BD FACSCount (BD Biosciences)			BD FACSCalibur (BD Biosciences)			BD FACSCount (BD Biosciences)	
**Hematology**	Ac·T 5 diff CP (Beckman Coulter)				Ac·T 5 diff CP (Beckman Coulter)				
**Chemistry** **	COBAS Integra 400 (Roche Diagnostics)				COBAS Integra 400 (Roche Diagnostics)				
**Hepatitis B antigen**	Murex HBsAg version 3 (Abbott Murex)				Murex HBsAg version 3 (Abbott Murex)				
**Hepatitis B antibody**	Murex antiHBs (Murex Biotech)				Murex antiHBs (Murex Biotech)				
**Syphilis** † ‡	Immutrep RPR (Omega Diagnostics)	BD Macro-Vue RPR (BD diagnostics)	Human RPR (Human Diagnostics)		Human RPR (Human Diagnostics)				
	Immutrep TPHA (Omega Diagnostics)	Randox TPHA (Randox Laboratories)	Human TPHA Liquid (Human Diagnostics)		Human TPHA Liquid (Human Diagnostics)			Hexagon (Human Diagnostics)	
***N. gonorrhoeae*** ***C. trachomatis***	APTIMA Combo 2 (Gen-Probe)				APTIMA Combo 2 (Gen-Probe)			COBAS Amplicor (Roche Diagnostics)	
***T. vaginalis***	APTIMA TV TMA (Gen-Probe)				APTIMA TV TMA (Gen-Probe)			In Pouch TV (Biomed Diagnostics)	

*Jinja & Kampala used Vironostika 3^rd^ gen until March 2010.

**Tororo & Mbale used Roche C111 until Sept 2010.

Kabwohe used ^†^Immutrep RPR, and

‡ Determine Syphilis TP until May 2010.

### Data collection and analysis

Demographic, behavioral, and clinical data were entered onto standard case report forms, which were scanned using intelligent character recognition (ICR) DataFax software (Clinical DataFax Systems Inc., Hamilton, Canada) and double-verified by independent data technicians. Data analyses were conducted using SAS version 9.2 (SAS Institute, Cary, NC).

## Results

### Demographic and sexual risk behavior characteristics

From July 2008 to November 2010, a total of 7920 HIV-1 serodiscordant couples were screened for study eligibility, and 4758 were enrolled, for a screen-to-enroll ratio of 1.7∶1. For 62% of enrolled couples, the HIV-1 susceptible partner was male (Table 3), and 56% of the couples were from Uganda. Although 98% of couples were married, with a median duration of the partnership of 7.0 years (interquartile range [IQR] 3.0–14.0), most couples had recently learned of their HIV-1 serodiscordant status [median 0.4 years (IQR 0.1–2.0)]. A minority of couples (n = 331, 6%) had previously participated in another HIV-1 prevention clinical trial (Partners in Prevention HSV/HIV Transmission Study) conducted at 5 of the 9 research sites [14].

**Table 3:** Baseline Characteristics of Enrolled HIV-1 Serodiscordant Couples (N = 4758).

*Median (interquartile range) or N (%)*				
	Couples with HIV-1 susceptible women (N = 1792)		Couples with HIV-1 susceptible men (N = 2966)	
	*HIV-1 infected male*	*HIV-1 susceptible female*	*HIV-1 infected female*	*HIV-1 susceptible male*
**Demographic Characteristics**				
Age, years	39 (33–44)	33 (28–38)	29 (24–35)	34 (28–41)
18–24	34 (2%)	232 (13%)	794 (27%)	300 (10%)
25–34	498 (28%)	817 (46%)	1426 (48%)	1285 (43%)
35–44	822 (46%)	596 (33%)	626 (21%)	902 (30%)
≥45	438 (24%)	147 (8%)	120 (4%)	479 (16%)
Education, years	7 (4–10)	6 (3–8)	7 (4–8)	8 (5–11)
Monthly income, any	1578 (88%)	1247 (70%)	1633 (55%)	2532 (85%)
**Couple Characteristics**				
Uganda (vs. Kenya)	1207 (67%)		1453 (49%)	
Married to study partner	1767 (99%)		2879 (97%)	
Living with study partner	1763 (98%)		2897 (98%)	
Years living with study partner	11 (5–18)		5 (2–10)	
Number of children together	3 (1–5)		1 (0–3)	
Years aware of HIV-1 serodiscordant status	0.7 (0.1–2.1)		0.3 (0.1–1.8)	
**Sexual Risk Behavior**				
Number of sex acts in prior month	4 (2–8)		4 (3–8)	
Any unprotected sex acts in prior month	408 (23%)		862 (29%)	
Any sex with outside partner in prior month	269 (15%)	10 (1%)	31 (1%)	406 (14%)
**Clinical Characteristics**				
CD4 cell count/µL	457 (354–596)	N/A	529 (396–704)	N/A
HIV-1 plasma RNA, log_10_ copies/mL	4.17 (3.51–4.74)	N/A	3.79 (3.20–4.38)	N/A
WHO Stage 1	1048 (58%)	N/A	1975 (67%)	N/A
Stage 2	589 (33%)	N/A	832 (28%)	N/A
Stage 3	155 (9%)	N/A	159 (5%)	N/A
Circumcised (men only)	579 (32%)	N/A	N/A	1580 (53%)
Using contraception*	N/A	841 (47%)	936 (32%)	N/A
Pregnant	N/A	0 (0%)	415 (14%)	N/A
**Sexually Transmitted Infections** **				
*N. gonorrhoeae*	1% (13/1688)	1% (21/1530)	2% (47/2568)	1% (21/2794)
*C. trachomatis*	<1% (7/1687)	1% (17/1530)	1% (19/2566)	2% (42/2799)
*T. vaginalis*	2% (28/1696)	7% (102/1612)	9% (251/2653)	3% (98/2807)
Syphilis (positive RPR & confirmatory test)	7% (117/1740)	6% (104/1737)	4% (115/2875)	4% (104/2879)

*Any contraceptive use includes: oral, injectable and implantable contraceptives, intrauterine device, hysterectomy or bilateral tubal ligation.

**Data on sexually transmitted infections available from a subset of participants.

The median age of HIV-1 susceptible participants was 33 years (IQR 28–40). HIV-1 susceptible partners reported a median of 4 sex acts (IQR 3–8) with their HIV-1 infected partner in the month before enrollment, and 27% reported sex unprotected by condoms with their study partner. At enrollment, 14% of HIV-1 susceptible men reported sex with an outside partner in the month prior to enrollment compared to 1% of women. Forty-seven percent of HIV-1 susceptible women used contraception, most of which was hormonal. At enrollment, 53% of HIV-1 susceptible men were circumcised.

The median age of HIV-1 infected participants was 33 years (IQR 26–39). The majority (64%) had WHO stage 1 HIV-1 disease, and 14% of HIV-1 infected women were pregnant at enrollment. The median baseline CD4 count was 496 cells/µL (IQR 375–662), and 81% had CD4 counts >350 cells/µL. The median HIV-1 plasma RNA level was 3.94 log_10_ copies/mL (IQR 3.31–4.53). HIV-1 plasma RNA concentrations were significantly higher in HIV-1 infected men versus women (median 4.17 log_10_ copies/mL verses 3.79 log_10_ copies/mL respectively; Mann-Whitney-Wilcoxon p<0.0001). One-quarter of HIV-1 infected partners had HIV-1 RNA <2000 copies/mL, and 19% had HIV-1 RNA >50,000 copies/mL.

The baseline prevalence of sexually transmitted infections (STIs) was low. *T. vaginalis* was the most prevalent STI and was more commonly detected in women (7% in HIV-1 susceptible and 9% in HIV-1 infected women) than in men (3% in HIV-1 susceptible and 2% in HIV-1 infected men).

Of 3162 HIV-1 serodiscordant couples who were screened for the study but did not enroll, 2912 (92%) were not eligible, 208 (7%) were eligible but did not enroll, and 42 (1%) did not complete screening. Major reasons for screening-out were CD4 count less than 250 cells/µL (51%) in the HIV-1 infected partners (thus meeting national criteria for antiretroviral therapy initiation), and pregnancy (23%), breastfeeding (1%), or chronic active hepatitis B infection (11%) among HIV-1 susceptible partners (Table 4). Less than 5% of couples screened out for creatinine elevation, glycosuria or proteinuria, which were exclusion criteria to minimize potential renal toxicity from tenofovir exposure. Overall, 47% of couples had more than one reason for exclusion.

**Table 4:** Reasons for Screening Out of Ineligible HIV-1 Serodiscordant Couples (N = 2912).

*Median (interquartile range) or N (%)*		
Characteristic	Couples with HIV-1 susceptible women (N = 1312)	Couples with HIV-1 susceptible men (N = 1600)
**Reasons for ineligibility (HIV-1 infected partner)**		
CD4<250 cells/µL	784 (60%)	706 (44%)
History of AIDS defining illness	16 (1%)	18 (1%)
On antiretroviral therapy	7 (1%)	52 (3%)
**Reasons for ineligibility (HIV-1 susceptible partner)**		
Pregnant or planning to become pregnant	66 (5%)	N/A
Breastfeeding	16 (1%)	N/A
Infected with hepatitis B virus (as determined by positive hepatitis B surface antigen)	97 (7%)	237 (15%)
Not meeting clinical study eligibility criteria*	35 (3%)	63 (4%)
Not meeting renal study eligibility criteria	60 (5%)	48 (3%)
Not meeting other laboratory criteria**	355 (27%)	454 (28%)
**Reasons for ineligibility (couple)**		
<6 sex acts with study partner in last 3 months	37 (3%)	63 (4%)
Planning to discontinue relationship	17 (1%)	39 (2%)
Planning to be away from study area >2 months during the study period	3 (<1%)	22 (1%)
Enrolled in other HIV-1 prevention or treatment trial	0 (0%)	0 (0%)
Other***	36 (3%)	63 (4%)

*Clinical exclusion criteria are detailed in Table 1.

**Other laboratory criteria are: total bilirubin, hepatic transaminases, absolute neutrophil count, platelets, and hemoglobin as detailed in Table 1.

***Other reason (n = 99 total) included: not meeting behavioral or administrative criteria (n = 48); not found to be HIV-1 serodiscordant upon testing at the study site (n = 26); and other clinical conditions/investigator decision (n = 25).

## Discussion

East African HIV-1 serodiscordant couples in which the HIV-1 infected partner did not meet ART eligibility by national guidelines, and who are at high risk of HIV-1 transmission, were successfully enrolled into the Partners PrEP Study, the largest efficacy trial of oral PrEP. The efficient accrual of nearly 4800 couples over a 28-month period, demonstrates the feasibility of identifying and recruiting a high-risk heterosexual population to determine the efficacy and safety of PrEP and other novel HIV-1 prevention interventions, and indicates that HIV-1 serodiscordant couples are readily identified in rural and urban settings in sub-Saharan Africa for targeted implementation and delivery of PrEP, if efficacious.

Plasma HIV-1 RNA concentrations are a primary determinant of the risk of transmission of HIV-1, which is highest when HIV-1 RNA concentrations are greater than 50,000 copies/mL [15], [16]; we observed these RNA levels at enrollment in 19% of HIV-1 infected partners in our cohort. Other biologic and behavioral correlates of sexual HIV-1 transmission include lack of male circumcision, younger age, and unprotected sex. Approximately one-third of HIV-1 susceptible partners were less than 30 years old, half of HIV-1 susceptible men were uncircumcised, and more than a quarter of couples reported sex unprotected by condoms. In our prior studies of HIV-1 serodiscordant couples, these baseline characteristics predicted higher HIV-1 incidence, in spite of behavioral change during prospective follow-up associated with ongoing risk-reduction counseling [14]. In recognition of these risks, Partners PrEP Study sites provide condoms, counseling about risk reduction, and referrals for HIV-1 susceptible uncircumcised men to male circumcision providers throughout follow-up. In long-term HIV-1 serodiscordant partnerships, the risk of transmission decreases over time, which may reflect biologic and behavioral factors [17], [18]. For HIV-1 prevention trials that require two to three years of intensive follow-up with a high retention rate, the inclusion criteria leads to selection of stable couples, as indicated by the median duration of partnership of seven years in the Partners PrEP Study.

Previous studies have reported the high rates of transmission of HIV-1 among serodiscordant couples who are unaware of their serostatus [15]. Couples counseling and mutual disclosure of serostatus have been temporally associated with increased condom use and lower HIV-1 incidence in HIV-1 serodiscordant couples [19], [20]. Although self-reported condom use at study enrollment was high in our cohort (73%), couples may have over-reported condom use, as 14% of HIV-1 infected women were pregnant at the time of study entry. Fertility rates are 4.9 and 6.7 per woman in Kenya and Uganda [21], and couples report social and cultural pressures to have children [22]. In settings where national guidelines limit provision of ART to those with CD4 counts less than 200 or 350 cells/µL, HIV-1 serodiscordant couples who desire children could potentially benefit from PrEP. At study entry, 14% of HIV-1 susceptible men reported outside sexual partners; in our previous work based on viral sequencing, we found that about 30% of incident HIV-1 infections occurring within HIV-1 serodiscordant couples were acquired from outside the primary sexual partnership [14].

Approximately 15% of participants who screened out were hepatitis B surface antigen (HBsAg) positive or met renal exclusion criteria based on serum creatinine levels or proteinuria. Although initial safety data from the 1% tenofovir gel and oral FTC/TDF PrEP studies are encouraging, if tenofovir-based PrEP is shown to be safe and efficacious in ongoing trials, PrEP safety will subsequently need to be assessed in HIV-1 susceptible persons with chronic active hepatitis B infection or pre-existing renal dysfunction, as well as pregnant and breast-feeding women. Both TDF and FTC have potent activity against hepatitis B virus (HBV), and a small number of exacerbations (i.e., flares) of chronic liver disease have been reported after withdrawal of these medications in HIV-1 infected persons co-infected with HBV [23], [24]. Moreover, TDF treatment rarely has been associated with decreases in proximal renal tubular function in HIV-1 infected persons [25], and FTC requires dose adjustment for moderate renal dysfunction. A recent systematic review and meta-analysis of the renal safety of TDF in HIV-1 infected individuals found that TDF use was associated with a statistically significant, but modest loss of renal function (difference in creatinine clearance between TDF users and controls of 3.92 mL/min), but not with increased risk of severe proteinuria, hypophosphatemia or bone fractures [26]. The long-term safety of TDF among HIV-1 uninfected persons is unknown. If PrEP is implemented for the prevention of HIV-1, a significant proportion of HIV-1 susceptible individuals, including those with renal dysfunction and hepatitis B but also pregnant and breastfeeding women, may be excluded from receiving PrEP until bridging studies determine the safety of this HIV-1 prevention intervention in these populations, and studies of renal and bone safety of long-term PrEP use will be important.

In conclusion, a cohort of almost 4800 East African heterosexual HIV-1 serodiscordant couples at high risk of HIV-1 transmission was efficiently recruited into a placebo-controlled efficacy trial of daily oral TDF and FTC/TDF PrEP for HIV-1 prevention. Given the high risk of HIV-1 transmission among serodiscordant couples, PrEP could be a cost-effective intervention if the efficacy is modestly high, the highest-risk couples can be targeted, and cost of delivery programs are comparable or lower than for antiretroviral treatment programs [27]. If PrEP is demonstrated to be safe and efficacious in the Partners PrEP Study and other ongoing PrEP trials, implementation of PrEP should be targeted to high-risk persons with normal renal function, including HIV-1 serodiscordant couples.

## References

[1] AVAC website. Oral and Topical PrEP Trials Timeline.. http://www.avac.org/prep.

[2] Tsai CC, Follis KE, Sabo A, Beck TW, Grant RF (1995). Prevention of SIV infection in macaques by (R)-9-(2-phosphonylmethoxypropyl)adenine.. Science.

[3] Garcia-Lerma JG, Otten RA, Qari SH, Jackson E, Cong ME (2008). Prevention of rectal SHIV transmission in macaques by daily or intermittent prophylaxis with emtricitabine and tenofovir.. PLoS Med.

[4] Garcia-Lerma JG, Cong ME, Mitchell J, Youngpairoj AS, Zheng Q (2010). Intermittent prophylaxis with oral truvada protects macaques from rectal SHIV infection.. Science Translational Medicine.

[5] Parikh UM, Dobard C, Sharma S, Cong ME, Jia H (2009). Complete protection from repeated vaginal simian-human immunodeficiency virus exposures in macaques by a topical gel containing tenofovir alone or with emtricitabine.. Journal of Virology.

[6] Subbarao S, Otten RA, Ramos A, Kim C, Jackson E (2006). Chemoprophylaxis with tenofovir disoproxil fumarate provided partial protection against infection with simian human immunodeficiency virus in macaques given multiple virus challenges.. J Infect Dis.

[7] Abdool Karim Q, Abdool Karim SS, Frohlich JA, Grobler AC, Baxter C (2010). Effectiveness and safety of tenofovir gel, an antiretroviral microbicide, for the prevention of HIV infection in women.. Science.

[8] Grant RM, Lama JR, Anderson PL, McMahan V, Liu AY (2010). Preexposure Chemoprophylaxis for HIV Prevention in Men Who Have Sex with Men.. N Engl J Med.

[9] FHI website. FHI to Initiate Orderly Closure of FEM-PrEP.. http://www.fhi.org/en/AboutFHI/Media/Releases/FEM-PrEP_statement041811.htm.

[10] Hugonnet S, Mosha F, Todd J, Mugeye K, Klokke A (2002). Incidence of HIV infection in stable sexual partnerships: a retrospective cohort study of 1802 couples in Mwanza Region, Tanzania.. J Acquir Immune Defic Syndr.

[11] Dunkle KL, Stephenson R, Karita E, Chomba E, Kayitenkore K (2008). New heterosexually transmitted HIV infections in married or cohabiting couples in urban Zambia and Rwanda: an analysis of survey and clinical data.. Lancet.

[12] Wabwire-Mangen F (2009). Uganda HIV Modes of Transmission and Prevention Response Analysis.

[13] Cohen MS, Chen YQ, McCauley M, Gamble T, Hosseinipour MC (2011). Prevention of HIV-1 infection with early antiretroviral therapy.. N Engl J Med.

[14] Celum C, Wald A, Lingappa JR, Magaret AS, Wang RS (2010). Acyclovir and Transmission of HIV-1 from Persons Infected with HIV-1 and HSV-2.. N Engl J Med.

[15] Quinn TC, Wawer MJ, Sewankambo N, Serwadda D, Li C (2000). Viral load and heterosexual transmission of human immunodeficiency virus type 1. Rakai Project Study Group.. N Engl J Med.

[16] Donnell D, Baeten JM, Kiarie J, Thomas KK, Stevens W (2010). Heterosexual HIV-1 transmission after initiation of antiretroviral therapy: a prospective cohort analysis.. Lancet.

[17] Shiboski SC, Padian NS (1998). Epidemiologic evidence for time variation in HIV infectivity.. J Acquir Immune Defic Syndr Hum Retrovirol.

[18] Vernazza PL, Eron JJ, Fiscus SA, Cohen MS (1999). Sexual transmission of HIV: infectiousness and prevention.. AIDS.

[19] Heffron R, Were E, Celum C, Mugo N, Ngure K (2010). A prospective study of contraceptive use among African women in HIV-1 serodiscordant partnerships.. Sex Transm Dis.

[20] Allen S, Meinzen-Derr J, Kautzman M, Zulu I, Trask S (2003). Sexual behavior of HIV discordant couples after HIV counseling and testing.. AIDS.

[21] Bongaarts J (2010). The Causes of Educational Differences in Fertility in sub-Saharan Africa..

[22] Nebie Y, Meda N, Leroy V, Mandelbrot L, Yaro S (2001). Sexual and reproductive life of women informed of their HIV seropositivity: a prospective cohort study in Burkina Faso.. Journal of Acquired Immune Deficiency Syndromes.

[23] Nuñez M, Soriano V (2005). Management of patients co-infected with hepatitis B virus and HIV.. Lancet Infectious Diseases.

[24] Lim SG, Wai CT, Rajnakova A, Kajiji T, Guan R (2002). Fatal hepatitis B reactivation following discontinuation of nucleoside analogues for chronic hepatitis B.. Gut.

[25] Gallant JE, Winston JA, DeJesus E, Pozniak AL, Chen SS (2008). The 3-year renal safety of a tenofovir disoproxil fumarate vs. a thymidine analogue-containing regimen in antiretroviral-naive patients.. AIDS.

[26] Cooper RD, Wiebe N, Smith N, Keiser P, Naicker S (2010). Systematic review and meta-analysis: renal safety of tenofovir disoproxil fumarate in HIV-infected patients.. Clin Infect Dis.

[27] Hallett T (2011). ART or PrEP for HIV Prevention in HIV Serodiscordant Partnerships: A Mathematical Modeling Comparison..

